# FLIM Reveals Red Light-Induced Changes in Murine Hair Follicles

**DOI:** 10.3390/bios16050232

**Published:** 2026-04-22

**Authors:** Shanjie Xu, Aoshan Wang, Yuxuan Lin, Qichang Lai, Guangchao Xu, Chunhua Peng, Xiao Peng, Wei Yan, Junle Qu

**Affiliations:** 1 State Key Laboratory of Radio Frequency Heterogeneous Integration (Shenzhen University), Key Laboratory of Optoelectronic Devices and Systems of Ministry of Education and Guangdong Province, College of Physics and Optoelectronic Engineering, Shenzhen University, Shenzhen 518060, China; 2410232022@mails.szu.edu.cn (S.X.); 2400233030@mails.szu.edu.cn (A.W.); 2022270023@mails.szu.edu.cn (Y.L.); 2022270259@mails.szu.edu.cn (Q.L.); jlqu@szu.edu.cn (J.Q.); 2 Guangdong Ashmore Health Technology Co., Ltd., Foshan 528305, China; 13802648486@163.com (G.X.); pch6@sina.com (C.P.)

**Keywords:** hair growth, red light irradiation, fluorescence lifetime imaging microscopy, skin tissue

## Abstract

Hair loss, particularly androgenetic alopecia (AGA) and alopecia areata (AA), is a prevalent condition with widespread psychosocial impact. Recently, low-level laser therapy (LLLT) has emerged as a promising non-invasive therapeutic alternative due to its bioregulatory effects and favorable safety profile compared to conventional pharmacological treatments. In this study, we employed fluorescence lifetime imaging microscopy (FLIM) to investigate the effects of red-light irradiation on hair follicle dynamics and the cutaneous microenvironment in a C57BL/6 mouse model. A hair regeneration model was established to evaluate the efficacy of 650 nm red-light irradiation (bandwidth ± 20 nm). Then, the skin tissue was stained with hematoxylin and eosin (H&E) and followed by FLIM analysis to provide a multidimensional assessment of tissue morphology and metabolic status. Results showed that red-light irradiation significantly increased hair follicle numbers and enhanced adenosine triphosphate (ATP) levels in the skin tissue. FLIM analysis further revealed prolonged fluorescence lifetime values across different epidermal and dermal layers in the irradiated group, indicating significant alterations in the skin metabolic microenvironment. Furthermore, phasor plot analysis enabled precise differentiation between hair follicles and their surrounding skin structures, highlighting FLIM’s high sensitivity and accuracy in evaluating hair growth. In conclusion, this study has provided novel imaging-based insights into the mechanisms of LLLT-induced hair regeneration, highlighting the potential of FLIM as a powerful tool for characterizing the cutaneous microenvironment and quantitatively evaluating phototherapeutic efficacy in future translational applications.

## 1. Introduction

Hair loss has emerged as a pervasive global concern, impacting individuals across all demographic strata. This rise is often associated with the synergy of genetic predisposition, occupational stress, and the emotional volatility characteristic of contemporary lifestyles. Among the diverse forms of alopecia, androgenetic alopecia (AGA) and alopecia areata (AA) are the most prevalent forms. AGA, commonly observed in men, is primarily attributable to genetic factors and androgenic activity, specifically dihydrotestosterone (DHT). In males, AGA typically presents as a recession of the frontal hairline and/or diffuse rarefaction at the crown, generally sparing the hair of the parietal and occipital regions [1]. Conversely, in females, AGA is characterized by diffuse hair thinning, primarily in the central and frontal scalp, with the frontal hairline typically remaining intact [2]. In contrast, AA is defined as an autoimmune disorder where the immune system initiates an erroneous assault on the hair follicles, resulting in localized circular patches of hair loss. Epidemiological data reports the incidence of AGA: approximately 30% of white males experience AGA by the age of 30, a figure that rises to 50% by the age of 50 [3]. In China, the prevalence of AGA among males in Shanghai is 19.9%, while in females it is 3.1% [4]. Across six other major Chinese metropolitan areas, the prevalence stands at 21.3% for men and 6.0% for women [5]. Taken together, these statistics underscore the urgent need for effective and well-tolerated therapeutic strategies to promote hair regrowth, particularly for male-pattern hair loss.

At present, minoxidil and finasteride are the only two drugs approved by the U.S. Food and Drug Administration (FDA) for treating AGA [6,7]. Specifically, minoxidil functions as a multifunctional biological response modulator that accelerates hair growth, induces dermal thickening, and prolongs the anagen (growth) phase of the hair cycle [8]. In contrast, finasteride, a 5α-reductase inhibitor, reduces the conversion of testosterone (T) into androgen dihydrotestosterone (DHT), thereby mitigating the progression of AGA [9]. Nevertheless, the application of these drugs is limited by significant constraints. Minoxidil may induce itching and local irritation, whereas finasteride is contraindicated in females, thus restricting its clinical utility. Consequently, the imperative to mitigate adverse effects has catalyzed substantial scholarly interest in alternative therapeutic modalities, such as low-level laser therapy (LLLT).

LLLT, also known as red light therapy, cold laser, soft laser, or photobiomodulation (PBM), is a form of optical therapy that utilizes continuous low-power density laser irradiation [10]. Most LLLT devices operate within the red to near-infrared wavelength range (600–1000 nm), employing a power density significantly below the threshold required to induce thermal tissue damage (10 mW/cm^2^) [11]. The mechanism of LLLT has been shown to accelerate hair growth through modulating human growth factors, reducing the number of follicles entering the catagen phase (regression phase), inducing leptin (a hormone involved in hair-cycle regulation) expression during the anagen phase (growth phase), and promoting perivascular angiogenesis via vascular endothelial growth factor-A (VEGF-A), leading to increased follicle diameter and accelerated hair regeneration [12,13,14]. Furthermore, research indicates that near-infrared wavelengths are absorbed by cytochrome c oxidase, a complex protein within cellular mitochondria. This activation enhances ATP synthesis and reduces oxidative stress while upregulating crucial signaling molecules such as nitric oxide (NO) and calcium ions (Ca^2+^) [15,16,17]. In addition to red light, other light-based therapies, such as blue light (420–480 nm) have been employed primarily to target hair loss associated with excessive sebum production, a common feature in AGA. Blue light serves to diminish sebaceous gland activity and prevents follicular blockage, thereby minimizing hair loss. However, blue light is rarely utilized as a standalone treatment for hair regrowth and is typically integrated with other modalities like laser or red-light therapy.

Hair growth is a dynamic, cyclical process consisting of three distinct phases: anagen, catagen, and telogen. The anagen phase is characterized by active hair shaft elongation and synthesis, whereas the subsequent catagen and telogen phases represent periods of follicular regression and rest [18]. Consequently, one of the crucial quantitative indicators for evaluating hair regrowth efficacy is the percentage of hair follicles in the anagen phase. The conventional and most widely adopted technique for this evaluation is hematoxylin and eosin (H&E) staining, which enhances the contrast of tissue structures and permits the visualization of morphological characteristics, including cell shape, distribution, and nuclear size [19,20]. Other histological techniques include immunohistochemical staining with Ki67 to quantify cellular proliferation and the Terminal deoxynucleotidyl transferase dUTP nick end labeling (TUNEL) assay to detect cellular apoptosis. However, conventional histological approaches are limited by their subjectivity, dependence on manual observation, and deficiency in quantitative precision. Moreover, complete serial sectioning and follicle enumeration are frequently required to obtain reliable results. Accordingly, complementary evaluation methods, such as scalp biopsy examination, hair root analysis, and global photography, are increasingly employed for hair loss evaluation [21].

To address the limitations of traditional histological evaluation, advanced imaging methods like FLIM are being implemented as complementary tools to enhance diagnostic accuracy. FLIM, a powerful, sophisticated technique often integrated with confocal laser scanning microscopy, allows for the detection of subtle microenvironmental changes associated with disease that are typically undetectable by conventional wide-field microscopy [22]. Fluorescence lifetime, independent of excitation intensity and fluorophore concentration under minimal quenching conditions, avoids inaccuracies arising from variations in fluorophore density and chemical degradation. However, photobleaching can still bias lifetime distributions (e.g., by preferential loss of long-lifetime components, leading to an apparent lifetime shortening) and should be minimized during acquisition [23]. Therefore, low laser power is usually used for imaging in FLIM experiments. Consequently, fluorescence lifetime serves as a reliable quantitative indicator of tissue biochemical environment, reflecting parameters such as viscosity, temperature, and polarity. FLIM has previously demonstrated its clinical utility in differentiating pathological and healthy tissues, particularly in cancer diagnostics [24]. Conventional H&E-stained sections remain the gold standard for evaluating hair growth efficacy because they provide clear visualization of tissue architecture and hair follicle morphology and are widely used in routine histopathological assessments. However, H&E staining is mainly limited to morphological evaluation and cannot fully reveal subtle microstructural, metabolic, or biochemical changes. Given its profound sensitivity to subtle microstructural and biochemical aberrations, even within conventionally prepared and stained tissue sections, FLIM has the potential to overcome the diagnostic limitations of H&E staining alone.

In this study, we have explored FLIM as a complementary technique in conventional histological analysis for the precise evaluation of hair follicle dynamics. Through the integration of FLIM with standard H&E staining, the biochemical property was obtained in assessing hair follicle structure and activity. This approach provided statistical data of the tissue status, establishing a quantitative evaluation platform of the therapeutic efficacy of LLLT in promoting hair regeneration.

## 2. Materials and Methods

### 2.1. Animal Models

**Figure 1:**
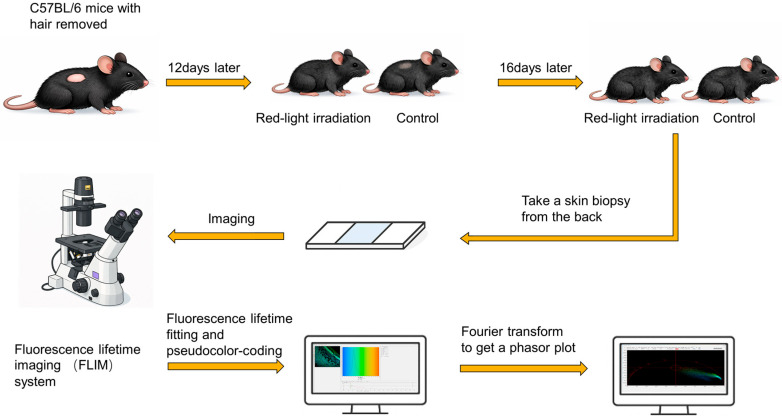
Schematic diagram of the workflow for analysis of dorsal skin tissue with FLIM technology.

Twelve male C57BL/6 mice (8 weeks old) were acquired for the experiment. To establish hair cycle synchronicity, the dorsal hair was removed using a depilatory cream over an area of approximately 2 cm × 2 cm. The mice were then randomly divided into two groups (n = 6 per group): the Red-Light Irradiation group and the Control group. Mice in the Red-Light Irradiation group received daily exposure to pulsed laser light with wavelength 650 ± 20 nm (AS-SF03, Ashmore Health Technology Co., Ltd., Foshan, China) for 10 min, following induction of anesthesia via isoflurane inhalation. The irradiation light used in this study was non-polarized, and no additional optical components were applied to control the polarization state during treatment. The Control group received the identical 10 min daily regimen of isoflurane anesthesia exposure, but without laser irradiation. Thermal imaging was conducted to confirm the safety of the treatment (see Figure S1). Photographs of each mouse were captured using a digital camera (Fujifilm, Tokyo, Japan) on days 0, 8, 12, and 16 following depilation to track hair regrowth (Figure 1).

### 2.2. Sample Preparation

To further assess the effects of red light on hair regeneration, dorsal skin tissue specimens were collected from both groups on day 16 of treatment. Tissues were fixed in 4% paraformaldehyde, embedded in paraffin, and sectioned for H&E staining. For ATP quantification, homogenized tissues were centrifuged in accordance with the specified tissue-to-lysis buffer ratio to yield the supernatant. The resulting supernatant was then mixed with the pre-prepared ATP assay working solution, and the relative light units (RLU) were rapidly measured using a chemiluminometry or liquid scintillation counter (Beyotime Biotechnology, Shanghai, China) to calculate the ATP concentration.

### 2.3. FLIM Performance Data Acquisition

Following the preparation of H&E-stained sections, bright-field imaging was initially conducted using a microscope (TS2-S-SM, Nikon, Tokyo, Japan) fitted with a 10× objective lens (oil immersion, NA 1.40, Nikon, Japan) to optimize the field of view. Representative images of skin morphology were acquired by manual adjustment of the stage position. FLIM was subsequently performed utilizing a FLIM system (DCS-120, Becker & Hickl GmbH, Germany) integrated with a confocal microscope (TS2-S-SM, Nikon, Tokyo, Japan) to acquire lifetime differences across tissue structures. A supercontinuum white laser (SC-PRO, YSL Inc., Wuhan, China) was used as the excitation source, which when combined with an AOTF (acousto-optical tunable filter, YSL Inc., Wuhan, China) can achieve arbitrary wavelength selection. The pulse width of laser is 6 ps, with 40 MHz frequency. Images were scanned at a resolution of 256 × 256 pixels and a rapid acquisition rate of up to 30 frames per second, with a high-speed Galvo Scanner (Cambridge Technology Inc., Bedford, MA, USA) for scanning. Time-correlated single photon counting (TCSPC) (SPC150, Becker & Hickl GmbH, Berlin, Germany) was achieved using a fluorescence lifetime imaging system, and single detection was achieved using a high-sensitivity hybrid photodetector (HPM-100-40, Becker & Hickl GmbH, Germany). Based on previously published literature [25,26,27] and preliminary experimental validation, the excitation and emission wavelengths were selected to optimize photon count acquisition. In this experiment, the excitation wavelength was set at 540 nm. To eliminate interference from excitation light, a 620/60 nm bandpass filter was installed in the emission channel.

### 2.4. Fluorescence Lifetime Fitting and Phasor Plot Analysis

After imaging, the FLIM data were processed using SPCImage NG software (version 7.4, Becker & Hickl GmbH), which integrates both time-domain and frequency-domain analyses and accommodates various decay models. The maximum likelihood estimation algorithm was employed to fit the fluorescence decay curve for each pixel. In addition to time-domain fitting, frequency-domain phasor plot analysis was executed to extract additional information [28]. Since eosin fluorescence exhibits biexponential decay behavior, the component number was set to two, and fitting parameters were calibrated to ensure precise alignment between the fitted curves and experimental data points. Because FLIM was performed on H&E-stained sections under the selected spectral window, the measured lifetime was primarily attributed to eosin fluorescence. Thus, lifetime shifts reported here mainly reflect changes in eosin’s microenvironment state [26]. The resulting fluorescence lifetime distributions were calculated, and an appropriate range (100–500 ps) was chosen for pseudo-color mapping. Color coding from blue to red was used to represent increasing lifetime values, enabling visualization of spatial lifetime differences across tissue structures (Figure 1). To further extract lifetime features, the phasor plot applied a Fourier transform to convert each pixel’s time-domain data into phase and modulation values in polar coordinates [29]. For each pixel, the TCSPC decay I(t) was converted to phasor coordinates by taking the first harmonic of the Fourier transform at angular frequency ω=2πf (where f is the laser repetition rate). The phasor components were computed as

$$g=\frac{\int_{0}^{T}I(t)cos(\omega t)dt}{\int_{0}^{T}I(t)dt},s=\frac{\int_{0}^{T}I(t)sin(\omega t)dt}{\int_{0}^{T}I(t)dt},$$

where T=1/f. In practice, the integrals were evaluated as discrete sums over TCSPC time bins. Pixels with similar decay signatures cluster in $\left(g,s\right)$ space and can be mapped back to their corresponding spatial locations for segmentation. Lifetime clusters were selected using region-of-interest (ROI) markers within the phasor map, allowing for the differentiation of regions characterized by similar lifetime values. These clusters were selectively highlighted in color, while unrelated regions remained uncolored, facilitating the clear distinction between different cell and tissue types.

This section was divided into subsections to provide a concise description of the experimental results and the respective interpretations.

## 3. Results

**Figure 2:**
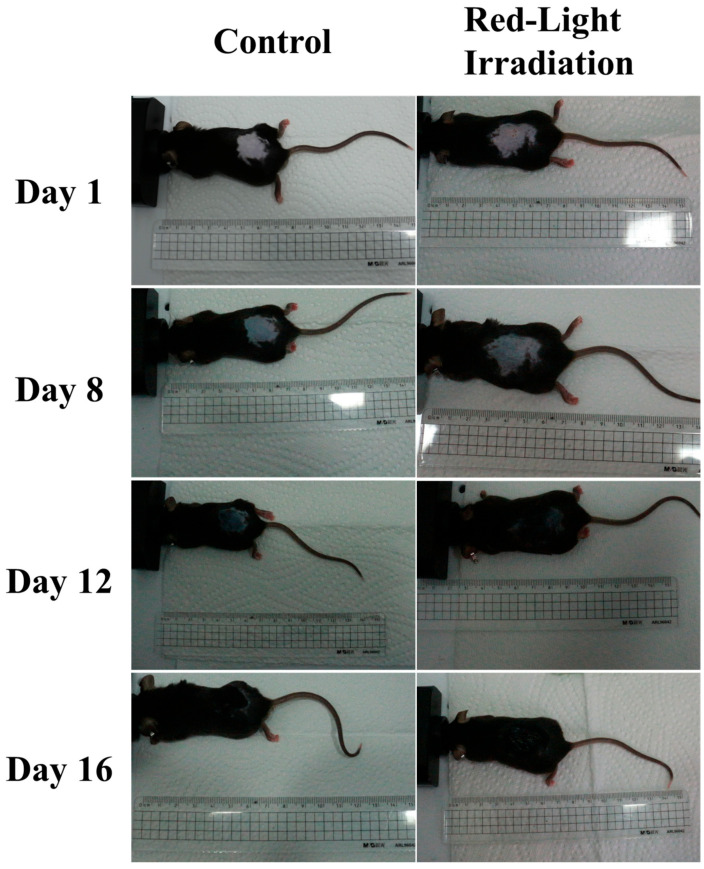
Macroscopic observation of hair growth in mice. Representative macroscopic images of the dorsal skin area of mice on days 1, 8, 12, and 16 post-depilation.

To assess the efficacy of red-light irradiation on hair growth, C57BL/6 mice received localized light treatment daily following depilation. On day 1, the skin in the depilated dorsal region of all mice appeared pink, indicating that the hair follicles were in the telogen phase of the hair cycle (Figure 2). By day 8, the skin color progressively changed from pink to grayish-black and eventually to black in all mice (Figure 2), reflecting the progression of hair follicles into the active anagen phase. On day 16, dorsal skin tissue samples from the treated area were collected for ATP concentration analysis.

**Figure 3:**
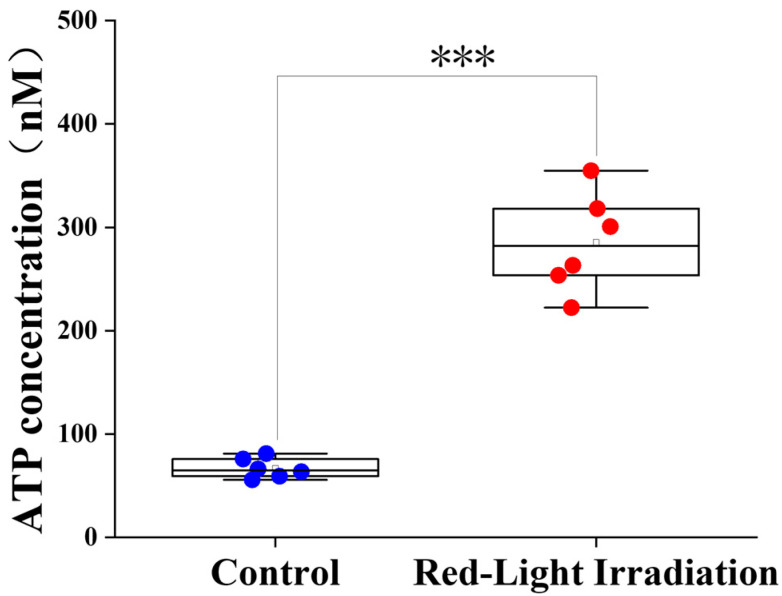
Biochemical analysis in mice. Comparison of ATP concentrations between the Control and Red-Light Irradiation groups. *** *p* < 0.001.

The Red-Light Irradiation group exhibited a significantly elevated ATP concentration in the skin tissue (mean ± SD: 285.42 ± 48.18 nM) compared to the Control group (66.91 ± 9.73 nM), reaching statistical significance (*** *p* < 0.001) (Figure 3). This enhanced ATP synthesis is consistent with the established mechanism of LLLT, which involves LLLT activating cytochrome c oxidase and enhancing mitochondrial electron transport. This leads to increased ATP levels, subsequently triggering the transition of hair follicles from telogen to anagen [10,11,12,13,14,15,17]. Previous experiments have demonstrated that in a mouse model of UV-induced photoaging, near-infrared (NIR) therapy resulted in increased ATP synthesis [16]. These results suggest that, compared with the Control group, the irradiated mice exhibited more evident hair regrowth and significantly higher ATP levels in the skin.

**Figure 4:**
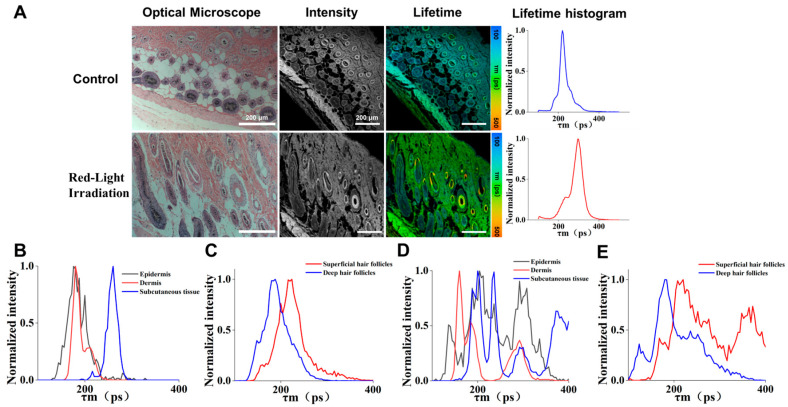
Bright-field and FLIM characterization of H&E-stained dorsal skin sections. “Control” refers to the group that received the same handling and anesthesia without irradiation, and “Red-Light Irradiation” refers to the group that received the same handling but was irradiated with a wavelength of 650 ± 20 nm. (**A**) Representative bright-field (H&E) images were acquired under the conventional microscope, while fluorescence intensity and lifetime images were acquired using a FLIM confocal system (DCS-120, Becker & Hickl, GmbH, Germany) (540 nm excitation; 620/60 nm emission), together with lifetime distribution histograms. Under these settings and our fitting model, the detected lifetime signal primarily originated from eosin fluorescence. (**B**,**C**) Normalized fluorescence lifetime intensities of different skin layers and hair follicles in the Control group. (**D**,**E**) Normalized fluorescence lifetime intensities of different skin layers and hair follicles in the Red-Light Irradiation group. Scale bar = 200 μm.

To assess these structural and biochemical alterations quantitatively, we utilized FLIM, a non-invasive technique that measures the decay rate of excited fluorophores, which is highly sensitive to the local cellular microenvironment [22,23,24,25,26]. This capability allows FLIM to provide a quantitative readout of fluorophore microenvironmental changes in the tissue, prompting us to analyze the H&E-stained skin tissue sections from mice (Figure 4). In this study, red light irradiation led to enhanced metabolic activity, which can be confirmed by ATP detection results. It also proved that the hair follicle microenvironment changed after red light irradiation, which was consistent with the conclusion obtained from the change in the fluorescence lifetime of FLIM. Comparing the bright-field, fluorescence intensity, and fluorescence lifetime images obtained revealed that, in contrast to the Control group, hair follicles in the Red-Light Irradiation group were converted from telogen to anagen, and the dermis was noticeably thickened. Moreover, the overall fluorescence lifetime was markedly increased (Figure 4A; more data in Figure S2), indicating that red light exposure had a significant impact on the microenvironment of the skin tissue.

A subsequent statistical analysis was performed on multiple skin compartments, including the epidermis, dermis, subcutaneous tissue, and superficial (upper dermal) and deep (lower dermal/subcutaneous) hair follicles. ROI were manually selected in the SPCImage software (version 7.4, Becker & Hickl GmbH) to obtain fluorescence decay curves. To ensure a fair comparison, fluorescence lifetime data from different tissue areas were normalized. (Figure 4B–E; further details in Figure S3). Across the diverse skin layers and hair follicle structures, the Red-Light Irradiation group exhibited significantly modulated fluorescence lifetimes compared to the Control group, characterized by layer-specific and statistically distinct changes. These lifetime shifts reflected alterations in the tissue microenvironment and further confirm the pronounced effect of light treatment on hair follicle growth.

**Figure 5:**
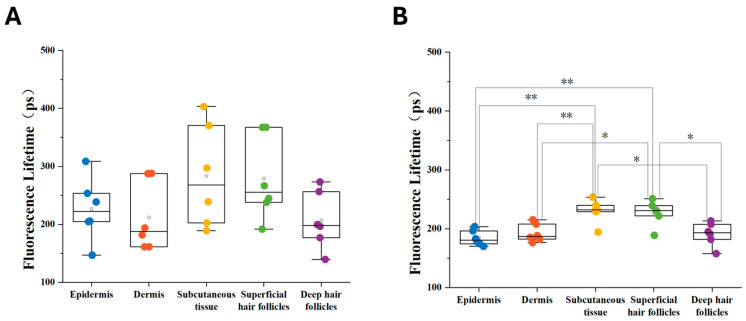
Quantitative statistical analysis of fluorescence lifetime across different skin layers and hair follicles. Under the selected excitation/emission conditions for H&E-FLIM, the measured fluorescence lifetime signal was primarily attributed to eosin fluorescence. Each dot represents one mouse (mouse-level mean calculated by averaging 3 ROIs per compartment; n = 6 mice/group). Boxplots were standardized (median/IQR; 1.5 × IQR whiskers), and statistical comparisons were performed on mouse-level values. Statistical significance in panel B was assessed using two-tailed unpaired two-sample *t*-tests. Comprehensive comparison of mean fluorescence lifetimes between (**A**) the Control group and (**B**) the Red-Light Irradiation group. * *p* < 0.05, ** *p* < 0.01. Approximate depth from the skin surface: epidermis (~0–0.05 mm), dermis (~0.05–0.5 mm), Subcutaneous tissue (~0.5–1 mm).

For quantitative analysis, the average fluorescence lifetimes of the selected ROIs were calculated to generate lifetime distribution histograms (Figure 5). The analysis revealed statistical significance in fluorescence lifetimes across skin layers and hair follicles. In the Control group, fluorescence lifetimes of superficial hair follicles were significantly higher than those of the epidermis (** *p* < 0.01), with a distinct divergence observed compared to dermis and deep follicles (* *p* < 0.05). Following the Red-Light Irradiation group, an overall upward shift in mean fluorescence lifetimes was noted, underscoring the modulation of the tissue microenvironment by LLLT. This comparison between superficial and deep hair follicles demonstrates that 650 nm light treatment affects the skin’s metabolism differently depending on the tissue depth. Furthermore, the overall upward shift in mean follicular lifetimes is consistent with the biological effects of LLLT on hair-follicle activation and anagen transition. Although changes in FLIM cannot serve as direct evidence of mitochondrial function and metabolic activation, they can provide corroborating evidence that mitochondrial function and metabolic activation lead to changes in the mitochondrial microenvironment.

**Figure 6:**
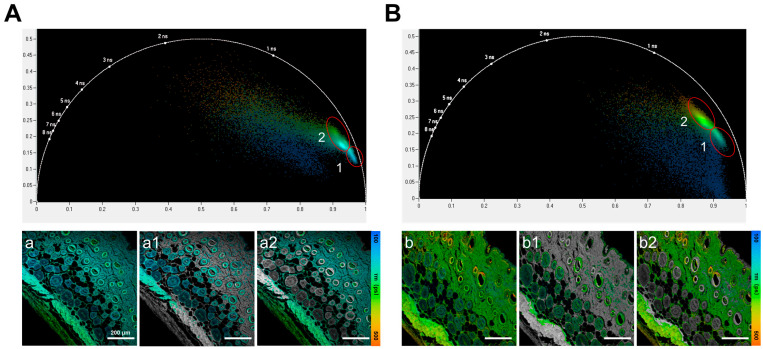
Phasor plot analysis of fluorescence lifetime images. The highlighted regions in the phasor plot represent ROI-based cluster selections used to group pixels with similar fluorescence lifetime signatures and map them back to their corresponding spatial locations. (**A**) Phasor plot of the Control group. (**a**) Overall fluorescence lifetime image; (**a1**,**a2**) corresponds to Clusters 1 and 2, respectively. (**B**) Phasor plot of the Red-light Irradiation group. (**b**) Overall fluorescence lifetime image; (**b1**,**b2**) corresponds to Clusters 1 and 2, respectively.

The phasor plot was utilized to convert each pixel’s time-domain data into phase and modulation values using a Fourier transform. Regions with similar lifetime values were then grouped into lifetime clusters within the phasor map, allowing for a clear, color-coded distinction between different cell and tissue types [29,30]. Phasor analysis provides a model-free clustering framework that can separate tissue components even when their mean lifetime ranges overlap, by leveraging differences in the overall lifetime decay signature. Importantly, cluster selection in the phasor plot was not performed arbitrarily. Following the standard phasor-FLIM workflow, clustered pixel populations were first identified in phasor space according to their distribution patterns and then mapped back to the original fluorescence lifetime image to verify their spatial correspondence and biological relevance [31,32]. Therefore, the final definition of each cluster was based on the combined interpretation of the phasor distribution and the corresponding image features, rather than on the phasor plot alone. Applying this clustering methodology, further analysis revealed the grouping of pixels with similar lifetimes into two main distinct clusters (Figure 6). These clusters corresponded to molecular components with different fluorescence lifetimes in the FLIM images. Specifically, Cluster 1 was associated with shorter-lifetime fluorophores, while Cluster 2 represented longer-lifetime components. Cluster 1 and Cluster 2 effectively differentiated between hair follicles and dermal and epidermal skin structures. These findings demonstrated that FLIM enables clear separation of both superficial and deep hair follicles from surrounding skin components in both the Control and Red-Light Irradiation groups, even when the lifetime differences are subtle. Because of the inter-animal variability, fluorescence lifetime differences were not interpreted as stand-alone evidence of treatment efficacy, but rather as complementary indicators of treatment-associated microenvironmental changes.

## 4. Discussion

While hair loss may not significantly compromise physiological function, its impact on self-esteem, social integration, and psychological resilience significantly diminishes the quality of life, thereby driving the sustained demand for effective treatment methods. LLLT, utilizing low-power-density red or near-infrared light, demonstrates significant efficacy in promoting cellular repair and stimulating blood circulation. Consequently, sustained scalp irradiation enhances metabolic activity within the skin and hair follicles, stimulating the proliferation of fibroblasts and hair follicle cells, thereby improving hair growth [10,11,12,13,14,15,16,17]. In this study, the effects of red-light irradiation on dorsal hair growth in C57BL/6 mice were systematically evaluated using a combination of H&E staining and FLIM technology. C57BL/6 mice serve as a commonly used model for hair growth and hair cycle research. At 6–8 weeks of age, all hair follicles in C57BL/6 mice enter the telogen phase; without intervention, they will not spontaneously transition back into the anagen phase [18]. In C57BL/6 mice, dorsal skin darkening is commonly used as a macroscopic indicator of anagen entry, associated with follicular melanogenesis [33]. Thus, C57BL/6 mice serve as an ideal model for investigating the functional transition of hair follicles from the telogen to the anagen phase under red light exposure.

Experimental findings revealed that, at the macroscopic level, mice in the Red-Light Irradiation group exhibited noticeable skin color changes starting on day 8 post-depilation. By day 16, the appearance of darker skin and visible hair regrowth was evident, indicating a successful transition of hair follicles from the telogen to anagen phase. ATP activity assays further supported this conclusion, with significantly higher ATP levels in the Red-Light Irradiation group compared to the Control group. This increase was consistent with enhanced cellular energy metabolism following red light therapy, which aligned with the established mechanism that red light activated the mitochondrial respiratory chain and increased ATP synthesis [15,16,17]. At the histological level, H&E staining revealed that red light exposure significantly increased both the number and diameter of hair follicles, while simultaneously inducing dermal thickening. This affirms that red light stimulated the transition of hair follicles into the growth phase and promoted structural remodeling of skin, as evidenced by dermal thickening and increased hair follicle number and diameter (Figure 4). Furthermore, changes in fluorescence lifetime revealed by FLIM technology provided more precise microenvironmental information. “Endogenous skin autofluorescence typically exhibits nanosecond-scale lifetimes (~1–3 ns) [34], whereas our H&E–FLIM had a lifetime range of picoseconds (100–500 ps), was dominated by eosin emission, and under our system’s spectral settings, the effect of autofluorescence on FLIM was negligible. Since hematoxylin does not fluoresce, the FRET effect in H&E sections causing changes of fluorescence lifetime can be ruled out. Therefore, the lifetime shifts are interpreted primarily as changes in eosin’s local microenvironment state [26]. In addition, molecular oxygen can act as a dynamic quencher in live tissues and may affect fluorescence lifetimes. However, our measurements were performed on fixed, paraffin-embedded H&E sections processed identically across groups, where in vivo oxygen gradients and diffusion states are not preserved. Therefore, the effect of oxygen concentration-related fluorescence quenching on fluorescence lifetime can be ignored. Given that the FLIM signal in H&E sections was dominated by eosin, the prolonged lifetime after red light treatment likely reflected red light–induced microenvironmental changes (e.g., polarity/pH/interactions) that altered eosin’s local environment and binding state [35,36]. However, these specific factors were not directly measured in this study and will be investigated in future work using controlled microenvironmental assays. Lifetime images and statistical analyses demonstrated that multiple skin structures, including both superficial and deep hair follicles, exhibited higher average fluorescence lifetime values in the Red-Light Irradiation group. This suggests red light exposure significantly alters tissue microenvironment conditions—such as polarity, pH, or intermolecular interactions—thereby supporting hair follicle regeneration. Furthermore, Phasor Plot analysis further strengthened the credibility of these findings. In both the Control and Red-Light Irradiation groups, the Phasor Plot clearly distinguished different lifetime clusters and precisely mapped corresponding structural regions in the images. Notably, in the Red-Light Irradiation group, Clusters 1 and 2 distinctly represented hair follicles and other skin structures, respectively, exhibiting more pronounced lifetime differences. This not only validates FLIM’s high sensitivity in tissues but also suggests that red light treatment exerts profound effects at the molecular level within tissues.

## 5. Conclusions

In conclusion, this study establishes a more precise and comprehensive methodology for evaluating hair growth by integrating FLIM with conventional H&E histological analysis. Through fluorescence lifetime measurements and phasor analysis based on the local microenvironment, this study highlights the utility of FLIM as a viable technique for investigating tissue changes associated with hair follicle growth in C57BL/6 mice. Concurrently, this approach provides quantitative, spatially resolved insights into the tissue microenvironment that cannot be obtained from conventional H&E brightfield images. The integration of Phasor–FLIM techniques with standard histological sections represents a promising, non-destructive quantitative assessment tool for elucidating hair growth mechanisms and phototherapy effects. By integrating additional optical indicators and molecular biomarkers, it holds significant potential to advance both clinical translation applications in hair regeneration and dermatological disease diagnosis and treatment.

## Data Availability

The data presented in this study are available on request from the corresponding author due to large amounts of data.

## Supplementary Materials

The following supporting information can be downloaded at: https://www.mdpi.com/article/10.3390/bios16050232/s1, Figure S1: Safety evaluation of 10-minute light irradiation. (**A**) Initial skin surface temperature measured by thermal imaging at the beginning of irradiation. (**B**) Skin surface temperature meas-ured by thermal imaging at the end of irradiation. Figure S2: Comparative imaging of skin sections. (**A**) Representative bright-field, fluorescence intensity, and fluorescence lifetime pseudo-colored images of the Control group (n = 6). (**B**) Representative bright-field, fluorescence intensity, and fluorescence lifetime pseudo-colored images of the Red-Light Irradiation group (n = 6). Figure S3: Fluorescence lifetime analysis of different skin components in each group of mice by randomly selecting three regions of interest (ROI) for each element. (**a**) Epidermis (**b**) Dermis (**c**) Subcutaneous tissue (**d**) Superficial hair follicle (**e**) Deep hair follicle.
