# Supplementary material for: FLIM Reveals Red Light-Induced Changes in Murine Hair Follicles
Source: Biosensors (Basel). 2026 Apr 22;16(5):232. doi: 10.3390/bios16050232 (PMC13204966; doi:10.3390/bios16050232)
Supplement: Supplementary file 1 [file biosensors-16-00232-s001.zip › biosensors-4165844-supplementary.pdf]

biosensors

MDPI

Supplementary Material

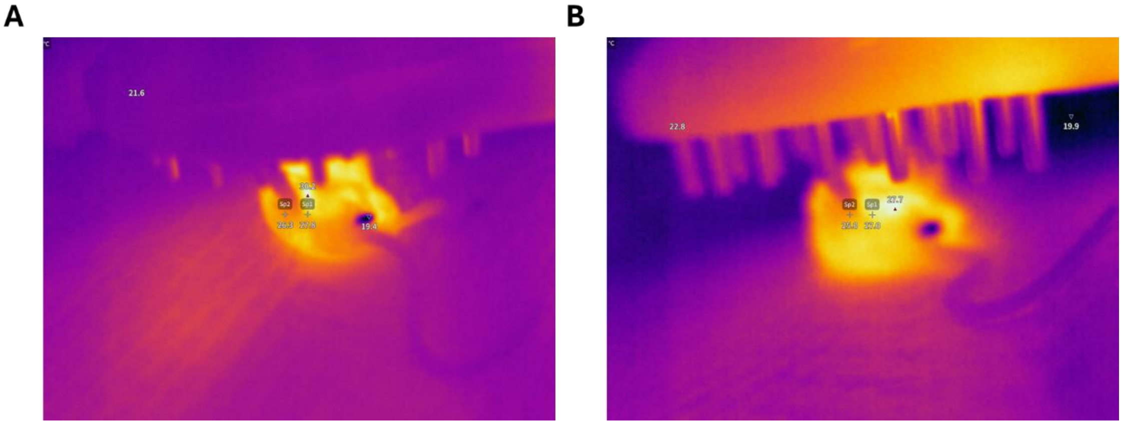

Figure S1. Safety evaluation of 10-minute light irradiation. (**A**) Initial skin surface temperature measured by thermal imaging at the beginning of irradiation. (**B**) Skin surface temperature measured by thermal imaging at the end of irradiation.

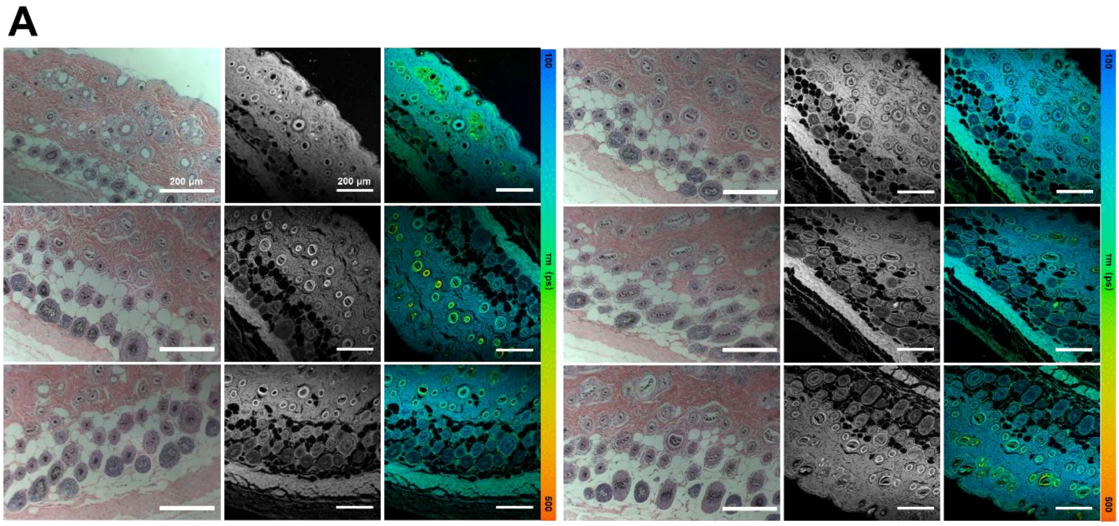

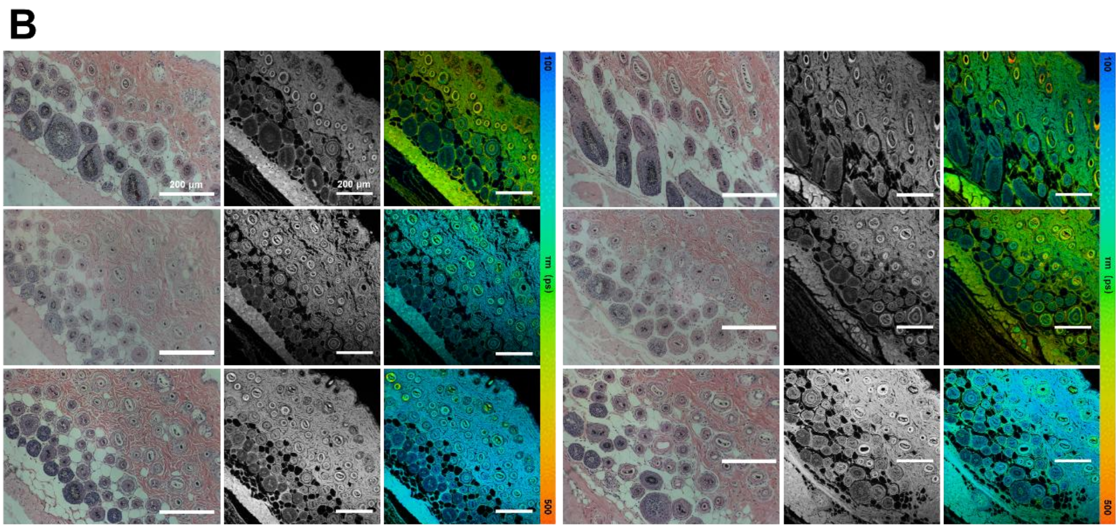

Figure S2. Comparative imaging of skin sections. (**A**)Representative bright-field, fluorescence intensity, and fluorescence lifetime pseudo-colored images of the Control group (n = 6). (**B**) Representative bright-field, fluorescence intensity, and fluorescence lifetime pseudo-colored images of the Red-Light Irradiation group (n = 6).

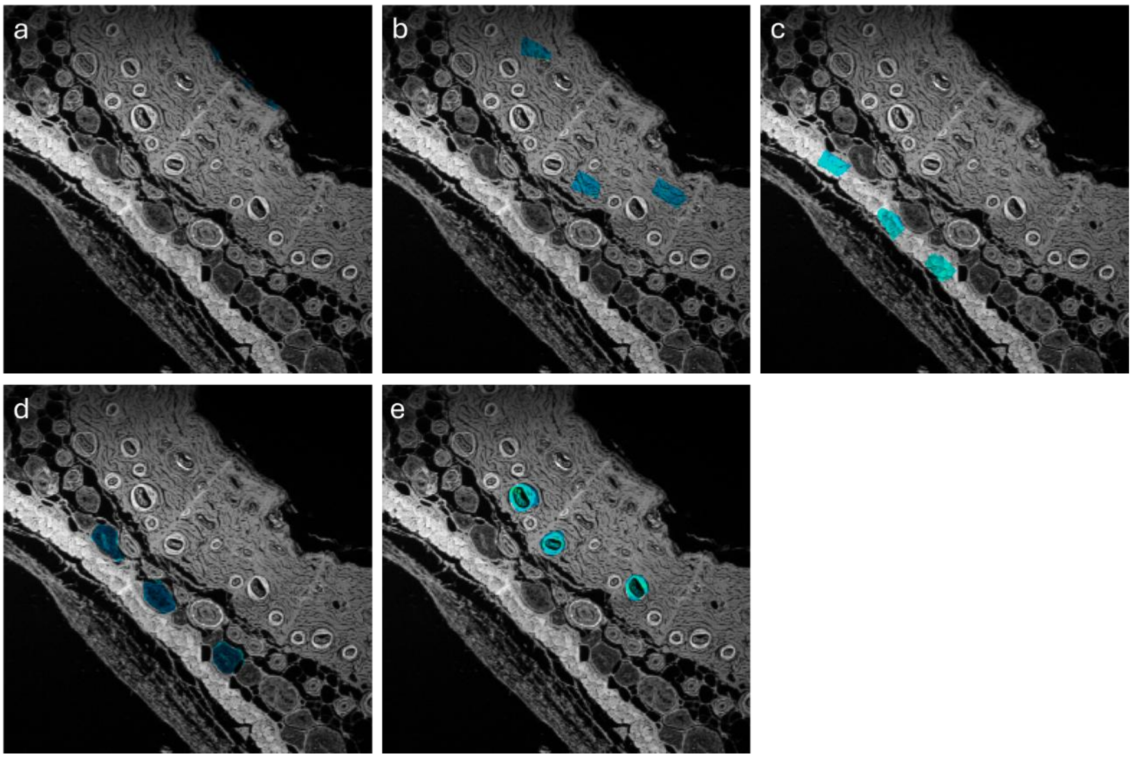

Figure S3. Fluorescence lifetime analysis of different skin components in each group of mice by randomly selecting three regions of interest (ROI) for each element. (**a**) Epidermis(**b**) Dermis(**c**) Subcutaneous tissue (**d**) Superficial hair follicle(**e**) Deep hair follicle.
